# Chromosome-Scale, Haplotype-Resolved Genome Assembly of *Suaeda Glauca*

**DOI:** 10.3389/fgene.2022.884081

**Published:** 2022-05-12

**Authors:** Liuxi Yi, Rula Sa, Shuwen Zhao, Xiaoming Zhang, Xudong Lu, Yingnan Mu, Siqin Bateer, Shaofeng Su, Shuyan Wang, Zhiwei Li, Shude Shi, Xiaoqing Zhao, Zhanyuan Lu

**Affiliations:** ^1^ Agricultural College, Inner Mongolia Agricultural University, Hohhot, China; ^2^ College of Grassland, Resources and Environment, Inner Mongolia Agricultural University, Hohhot, China; ^3^ School of Pharmaceutical Sciences, Baotou Medical College, Baotou, China; ^4^ Inner Mongolia Academy of Agricultural & Animal Husbandry Sciences/Inner Mongolia Key Laboratory of Degradation Farmland Ecological Restoration and Pollution Control/Inner Mongolia Conservation Tillage Engineering Technology Research Center, Hohhot, China

**Keywords:** haplotype-resolved assembly, genome assembly, Hi-C, HiFi, Suaeda glauca (bunge)

## Introduction

*Suaeda glauca* (Bunge) is an annual herb of the family Amaranthaceae (Wang et al., 2018; Qiu et al., 2021). It is widely distributed in the inland saline soil and seashore salt marsh of China, Mongolia, Siberia, Korea, and Japan (Duan et al., 2018; Qu et al., 2019). *Suaeda glauca* has a very high resistance to salt and alkali, and can survive well in soil environments with pH > 10 and salt content > 0.48% (Yang et al., 2008). It is widely used in windbreak sand fixation and soil improvement (Song and Wang, 2015). The succulent leaves of *Suaeda glauca* are edible and can be used as important pharmacological plant resources (Kefu et al., 2002) and can increase the number and volume of plant cells, which can absorb and store a large amount of water, thereby significantly increasing the water content per unit weight and volume of tissue, thus diluting the concentration of salt ions in leaf cells and maintaining the osmotic balance (Flowers and Colmer, 2008). It can effectively absorb heavy metal or salt in the soil and improve the soil environment (Bader et al., 2019). The evolved mechanism of *Suaeda glauca* for adapting to most adversity can provide precious materials and gene resources for the molecular biological study on the mechanism of plant stress resistance. In addition, the seeds of *Suaeda glauca* have high oil content and rich unsaturated fatty acids, which have extremely high development and utilization value as a potential special oil crop. It is reported that the oil content in the seeds accounts for 19% of the dry weight of the seeds, of which about 70% is linoleic acid (Zhao et al., 2018). *Suaeda glauca* extract can be developed into a prebiotic functional beverage (Song et al., 2019). Therefore, as a model halophyte containing a variety of nutrients, *Suaeda glauca* has received extensive attention in the area of medical, animal husbandry, food, industry, and salt tolerance gene development.

With the advent of the genome era, Beta vulgaris (Rodríguez del Río et al., 2019), *Spinacia oleracea* (Cai et al., 2021), and Chenopodium quinoa (Jarvis et al., 2017) of the Amaranthaceae plants, have been sequenced and assembled high-quality genomes. The evolution of the Amaranthaceae genomes is very complex, and their karyotypes and genome size differ greatly (Cai et al., 2021). Complete assemblies for *Suaeda glauca* mitochondria (474,330 bp) and chloroplast (149,807 bp) have been published (Qu et al., 2019; Cheng Y. et al., 2021). So far, the chromosome level genome of the genus *Suaeda* has not been reported. A high-quality reference genome will speed up the study of the stress resistance mechanism and functional genome of *Suaeda glauca*, as well as the evolutionary history of the Amaranthaceae genome.

In the present study, we used PacBio HiFi and Hi-C reads to assemble the *Suaeda glauca* genome, as well as annotated genes using transcriptome data from root, stem, leaf, and fruit. We believe this work will provide a scientific basis for molecular biology research, development, and utilization of *Suaeda glauca*.

## Materials and Methods

### Sample Collection

The *Suaeda glauca* used for this study was collected in Toketuo County (Supplementary Figure S1), Hohhot City, Inner Mongolia, China, located on the Tumochuan Plain. The geographical location is 111°40′91″ east longitude and 40°50′90″ north latitude. Mainly distributed salt vegetation types, the soil is saline-alkali land, the organic matter content is 4.12 g/kg, the hydrolyzable nitrogen is 179 mg/kg, the available phosphorus is 12.9 mg/kg, the available potassium is 124 mg/kg, the total amount of water-soluble salt is 24 g/kg, and the pH is 8.95. On July 9, 2021, we collected wild *Suaeda glauca* in this area. The plant height is 105 cm, green, the stem is erect, cylindrical, and the leaves are fleshy and semi-cylindrical. Fresh leaves were collected from the same plant for Hi-C and HiFi sequencing, and the roots, stems, leaves, and fruits of the plant were collected at the same time.

### DNA and RNA Extraction and Sequencing

For PacBio HiFi sequencing, high molecular weight genomic DNA was extracted and purified from leaves using Qiagen’s MagAttract HMW DNA Kit (QIAGEN, Germantown, MD, United States) per the manufacturer’s protocol. The resulting high molecular weight genomic DNA was then sheared to a target size of 15∼20 kb using the MegaRuptor 3 (Diagenode, Denville, NJ, United States). The HiFi sequencing library was prepared using SMRTbell Express Template Prep Kit 2.0 (Pacific Biosciences, Menlo Park, CA, United States) and followed by immediate treatment with the Enzyme Clean Up Kit (Pacific Biosciences, Menlo Park, CA, United States). Raw base-called data was processed to generate HiFi reads using the CCS program v4.2.0 (https://ccs.how/) with the following settings: minimum pass 3, minimum subread length 50, minimum predicted accuracy 0.99.

HiC library was prepared from young leaves, fixed using formaldehyde, and then lysed before the cross-linked DNA was digested overnight with DpnII. Sticky ends were biotinylated and proximity-ligated to form chimeric junctions that were enriched for and then physically sheared to a size of 300–500 bp. Chimeric fragments representing the original cross-linked long-distance physical interactions were processed into paired-end sequencing libraries. Paired-end 150 bp reads were generated using the Illumina NovaSeq 6000 sequencing system.

Total RNA was extracted from root, stem, leaf, and fruit using Trizol reagent (Invitrogen, CA, United States) and purified using an RNeasy Plant Mini Kit (Qiagen, CA, United States) according to the manufacturer’s instructions. RNA degradation and contamination were checked by 1% agarose gel electrophoresis. The quality and integrity of RNA were assessed using an Agilent Bioanalyzer 2100 system (Agilent, CA, United States); RNA Integrity Number (RIN) values were greater than 8.5 for all samples. After total RNA extraction, mRNA was enriched by Oligo (dT) beads. Sequencing libraries were prepared using an Illumina TruSeq RNA Library Preparation Kit (Illumina, CA, United States) as per the manufacturer’s protocol and sequenced on an Illumina HiSeq X platform, and 150 bp paired-end reads were generated.

### Genome Assembly

The HiFi reads were assembled using Hifiasm v0.13-r308 (Cheng H. et al., 2021). Integrated with the Hi-C reads, Hifiasm generated a primary and a pair of haplotype-resolved assemblies. The completeness of coding sequences of these assemblies was assessed using BUSCO v4.1.4 (Seppey et al., 2019). We used Minimap2 v2.17-r941 (Li, 2018, 2) to align the assembled contigs to the downloaded mitochondria (MW561632.1) and chloroplast (MK867773.1) sequences of *Suaeda glauca*. If 50% of a contig could be aligned, then the contig was removed. We aligned the HiFi reads to the primary assembly and found a small heterozygous peak below the depth of 5 × (Supplementary Figure S2). Then, purge_dups v1.2.5 (Guan et al., 2020) was used to remove redundant haplotigs. Finally, the 3D-DNA v180922 (Dudchenko et al., 2017) pipeline was used for anchoring contigs onto chromosomes and then manually polished using Juicebox Assembly Tools v1.11.08 (Dudchenko et al., 2018). The Hi-C interaction heatmap was generated using HiCExplorer (Wolff et al., 2020).

### Repetitive Element Annotation

We used the RepeatMasker v4.1.0 (Smit et al., 2019) program to identify repeat sequences. First, a customer repeat library was built using RepeatModeler v2.0.1 (Flynn et al., 2020), which first identified TEs *de novo* using RepeatScout v1.0.6 (Price et al., 2005) and RECON v1.08 (Bao and Eddy, 2002). Then, it used LTRharvest (Ellinghaus et al., 2008) and LTR_retriever v2.9.0 (Ou and Jiang, 2018) to discover high-quality LTR families. Finally, CD-HIT v4.8.1 (Li and Godzik, 2006) was used to remove the redundant TEs. These TE families were annotated and classified through the Dfam v3.1 (Hubley et al., 2016) and Repbase v20181026 (Bao et al., 2015) databases.

### Gene Structure Prediction and Functional Annotation

We performed gene structure prediction using the ETP model of the Brake2 pipeline v2.1.6 (Hoff et al., 2019; Brůna et al., 2021), which integrates homologous protein-based evidence and transcriptome evidence from four tissues of root, stem, leaf, and fruit. First, we used ProtHint (Brůna et al., 2020) to align the 3,510,742 green plant orthologous genes downloaded from OrthoDB v10.1 (Kriventseva et al., 2019) to the genome sequences after duplication masking. Meanwhile, RNA-Seq data were aligned to the duplicate-masked genome using HISAT2 v2.1.0 (Kim et al., 2015, 2). Then, GeneMark-ETP + uses evidence from these two sources to make initial unsupervised gene predictions. Finally, using the above initial predicted high-quality gene model, AUGUSTUS v3.4.0 (Stanke et al., 2006) was trained for two rounds. Combined with transcriptome and homologous protein evidence, the final gene structures were predicted using AUGUSTUS. GUSHR v1.0.0 (Gaius-Augustus/GUSHR, 2020) was used to predict UTRs utilizing coverage information from RNA-Seq data.

We used DIAMOND v2.0.9.147 (Buchfink et al., 2015) with parameter: moresensitive -p 64 -e 1e-6 –max-hsps 1 -k 1 -f 6 to align the annotated genes with the NR (RefSeq non-redundant proteins, 2020) and Swiss-Prot (Bairoch and Apweiler, 2000) databases, respectively. Using the eggNOG-mapper v2 (Huerta-Cepas et al., 2017) annotation pipeline, genes were annotated to the eggNOG 5.0 database (Huerta-Cepas et al., 2019). The InterProScan v5.50 (Jones et al., 2014) procedure was used for the PFAM database annotation (Mistry et al., 2021).

### Syntenic Analysis

For detecting syntenic blocks in the assembled genome, we first conducted an all-vs-all blast of primary protein sequences using DIAMOND with the parameters “--threads 80 --evalue 1e-6 --more-sensitive--outfmt 6”. Then, MCScanX (Wang et al., 2012) was used to identify collinear blocks. For pairwise synteny visualization of *Suaeda glauca* and *Beta vulgaris*, a Python version of MCScan implemented in jcvi (Tang et al., 2015) was used to align CDS sequences and plot.

### Gene Family Analysis

OrthoFinder v2.4.0 (Emms and Kelly, 2015) software was used for gene family clustering and species tree construction. Then the species tree was calibrated with the obtained branch lengths and calibration points obtained from TimeTree (Kumar et al., 2017) using r8s v1.8.1 (Sanderson, 2003). CAFE v4.2.1 (De Bie et al., 2006) was used to model the expansion and contraction of orthologous gene families.

### Assembly of the Chloroplast Genome

We aligned the HiFi reads to the published *Suaeda glauca* chloroplast genome using Minimap2, collected reads that 50% could be aligned, and assembled them using Canu v2.2 (Koren et al., 2017). Chloroplast gene prediction was using GeSeq (Tillich et al., 2017). The circular chloroplast gene annotation map was constructed using the OrganellarGenome DRAW tool (Lohse et al., 2013).

### Preliminary Data Analysis

**FIGURE 1:**
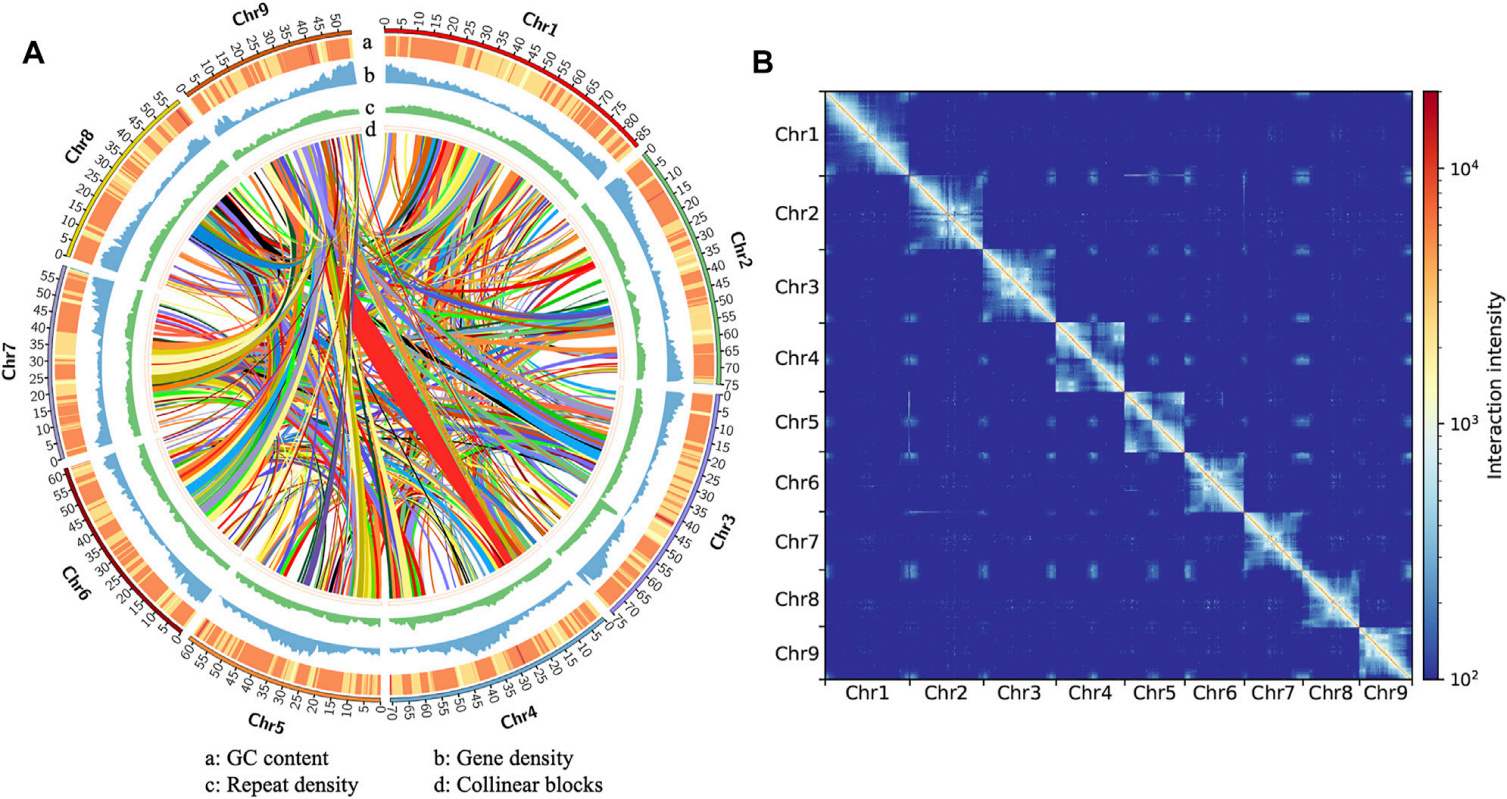
**(A)** Genomic features and collinear blocks across the *Suaeda glauca* genome assembly. The GC content, gene density and repeat density were calculated with a window size of 1 Mb and step size of 100 Kb. The colored bands in the circos plot indicated different syntenic blocks detected using proteins. **(B)** Genome-wide Hi-C interaction heatmaps at 500 Kb windows.

A total of 17.59 Gb HiFi data were generated, including 1,365,658 reads. The maximum length was 62.12 Kb, the average length was 12.88 Kb, and the N50 was 13.23 Kb (Supplementary Figure S3). For Hi-C sequencing, a total of 115.79 Gb of high-quality data with a Q20 ratio of 96.30% was obtained. The contig N50 of draft assemblies were 21.98, 6.52, and 6.06 Mb for the primary, haplotype 1, and haplotype 2 assemblies, spanning 664.20, 647.73, and 638.59 Mb, respectively, (Supplementary Table S1). We identified 94.4, 94.3, and 93.7% of eudicots conserved single copy homologous genes with the database of eudicots_odb10. Considering the integrity and continuity of the assemblies, we used the primary assembly for subsequent analysis. First, 41.25 Mb organelle sequences and redundant haplotigs were removed, and there was no significant change in the completeness of the BUSCO assessment (94.3%) after this process. Then, contigs were anchored onto chromosomes using a Hi-C contact map. The final assembly size was 622.95 Mb, consisting of 9 chromosomes, accounting for 96.79% of the total assembly size (Figure 1, Supplementary Table S1). A circular complete chloroplast genome of 149,811 bp was also assembled using HiFi reads (Supplementary Figure S4). The size was close to the published chloroplast genome (149,807 bp for MK867773.1), and the blast identity was 99.94%, including 51 gaps.

A customer repeat library containing 2,634 consensuses TEs was constructed. Through RepeatMasker, we identified 442.12 Mb repeats, accounting for 70.97% of the genome, which was much higher than the same genus *Suaeda aralocaspica* (38.41%). Among them, LTR is the most abundant, accounting for 37.11% of the genome (Figure 1A, Supplementary Figure S5A). This suggests that the *Suaeda glauca* genome is much larger than *Suaeda aralocaspica* may be caused by LTR insertions. We used the LTR_retriever procedure to estimate the insertion time of LTRs and found that there was a recent burst of LTR insertions in the *Suaeda glauca* genome compared with *Suaeda aralocaspica* (Supplementary Figure S5B).

For RNA-Seq, a total of 30.05 Gb of reads was generated, with an average of 7.51 Gb per tissue, and the average Q20 of the data was 97.51% (Supplementary Table S2). By integrating evidence from transcriptome and homologous proteins, a total of 32,503 genes and 34,039 transcripts were annotated. Of which, 26,657 (82.0%), 19,236 (59.2%), 22,290 (68.6%) and 19,583 (60.2%) genes were annotated with the NR, Swiss-Prot, eggNOG and Pfam databases separately. Ultimately, a total of 26,741 (82.3%) genes could be annotated to at least one database (Supplementary Table S3).

We used BUSCO to assess the assembly integrity of coding sequences (Supplementary Figure S6A). In the assembled sequence, 94.3% (2193 of 2326) of the dicot single-copy homologous genes in the eudicots_odb10 database were identified. Among the annotated genes, 94.1% (2190 of 2326) of the dicot single-copy homologous genes were identified. The LTR Assembly Index (LAI) index was used to assess the integrity of the repeated sequence assembly (Ou et al., 2018). The LAI score was calculated using the LTR_retriever pipeline. The raw LAI of the assembled sequence was 14.84, and the corrected LAI was 10.68 (Supplementary Figure S6B). We aligned HiFi reads to our assembled sequence, and 99.35% of the reads were aligned. These shreds of evidence demonstrate the high quality of our assembly.

**FIGURE 2:**
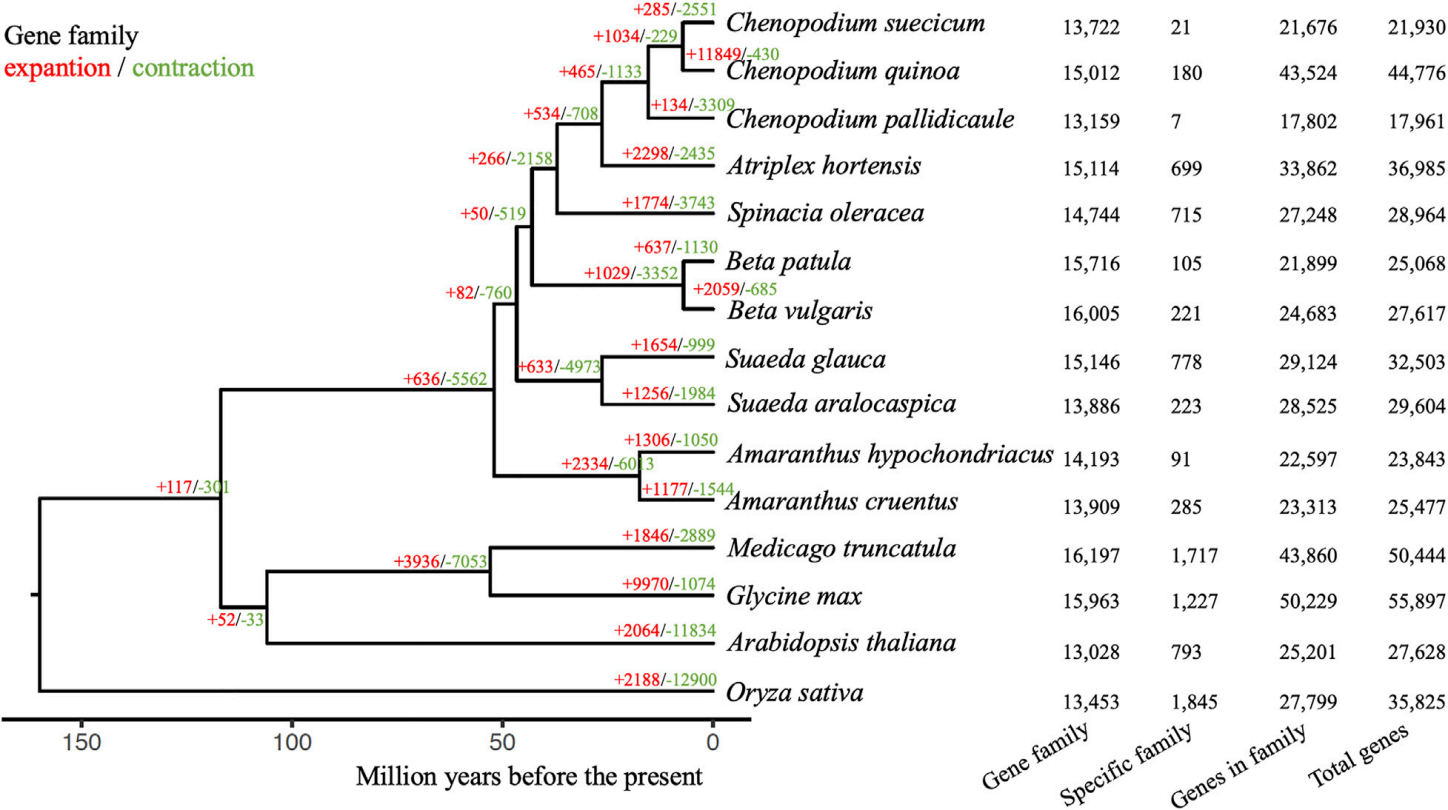
Gene family characteristics of *Suaeda glauca* and 14 other plants. The species tree was inferred using homologous proteins and calibrated with the obtained branch lengths and calibration points acquired from TimeTree. The red colored numbers indicated the numbers of expansion gene families and the green colored numbers indicated the number of contraction gene families. The number of gene families, specific family, genes in the family and total number of genes are shown on the right for each species.

Gene family analysis was performed for *Suaeda glauca* and other 14 plant species, including 10 Amaranthaceae (*Chenopodium suecicum, Chenopodium quinoa, Chenopodium pallidicaule, Atriplex hortensis, Spinacia oleracea, Beta patula, Beta vulgaris, Suaeda aralocaspica, Amaranthus hypochondriacus, Amaranthus cruentus*), *Medicago truncatula, Glycine max, Arabidopsis thaliana* and *Oryza sativa* as outgroup. A total of 31,299 gene families were identified, including 21 single-copy homologous gene families. 89.6% of *Suaeda glauca* genes can be assigned to orthogroups. Out of 15,146 orthogroups for *Suaeda glauca*, 778 gene families were specific to *Suaeda glauca* (Figure 2). 4,007 genes were contained in these gene families. Then GO and KEGG enrichment analyses were performed using clusterProfiler (Yu et al., 2012) (Supplementary Tables S4, S5). The phylogenetic tree shows that *Suaeda glauca* and *Suaeda aralocaspica* diverged at ∼26.36 million years ago (MYA), and Amaranthaceae species diverged began at ∼52.00 MYA. After differentiation, there were 1,654 gene families expanded, and 999 gene families contracted for *Suaeda glauca*. Of these, 169 gene families evolved rapidly (significantly higher than natural birth and death rates). We also performed GO and KEGG enrichment for these gene families (Supplementary Tables S6, S7). Some of the significantly enriched categories in the flax specific or rapidly evolving gene families may relate to unsaturated fatty acids metabolism (map00640: Propanoate metabolism, map01040: Biosynthesis of unsaturated fatty acids, map01212: Fatty acid metabolism), DNA repair (GO:0006281: DNA repair, GO:0006974: cellular response to DNA damage stimulus, GO:0006284: base-excision repair, GO:0045004: DNA replication proofreading, map03410: Base excision repair), and saline-alkaline tolerance [GO:0046686: response to cadmium ion, map04141: Protein processing in endoplasmic reticulum, map00020: Citrate cycle (TCA cycle)], which is related to the fact that *Suaeda glauca* is rich in linoleic acid and linolenic acid and grows in saline-alkali land.

## Conclusion

Using PacBio HiFi and Hi-C sequencing data, we successfully assembled chromosome-scale, haplotype-resolved assemblies of the *Suaeda glauca* genome. The size of the final primary assembly was 622.95 Mb and the contig N50 was 19.42 Mb, with a total of 211 contigs. By Hi-C sequencing, these contigs were anchored to nine chromosomes and accounted for 96.79% of the total assembly size. Repeated sequences accounted for 70.97% of the *Suaeda glauca* genome, mainly LTR. *Suaeda glauca* genome has a much higher proportion of repetitive sequences than the same genus *Suaeda aralocaspica*, and its genome size is also larger than that of *Suaeda aralocaspica*, which is presumed to be caused by a recent burst of LTR insertions. We annotated a total of 32,503 genes and 34,039 transcripts. The BUSCO-assessed completeness of the genome was 94.3%, and the BUSCO-assessed completeness of the annotated genes was 94.1%. The syntenic analysis showed that the genomes of *Suaeda glauca* and *Beta vulgaris* had high collinearity (Supplementary Figure S7). There were also many rearrangements between the two genomes, indicating assembly errors or true rearrangements, and more evidence is needed to prove it. Using HiFi data, we assembled the complete chloroplast genome of *Suaeda glauca* with a size of 149,811 bp. The gene family and phylogenetic tree analysis of *Suaeda glauca* and other ten species of Amaranthaceae showed that *Suaeda glauca* and *Suaeda aralocaspica* diverged at ∼26.36 MYA, and each Amaranthaceae species began to diverge at ∼52.00 MYA. To our knowledge, this is the first high-quality genome of *Suaeda glauca* and one of the most complete genomes of Amaranthaceae, laying the foundation for studying the evolution of Amaranthaceae species and the utilization of *Suaeda glauca*.

## Data Availability

The raw sequence data reported in this paper have been deposited in the Genome Sequence Archive (Chen et al., 2021) in National Genomics Data Center (CNCB-NGDC Members and Partners, 2022), China National Center for Bioinformation/Beijing Institute of Genomics, Chinese Academy of Sciences (GSA: CRA006064) that are publicly accessible at https://ngdc.cncb.ac.cn/gsa. The assembly and annotation files are deposited at the Zenodo (https://doi.org/10.5281/zenodo.6093785).
