# Supplementary figures and images for: Chromosome-Scale, Haplotype-Resolved Genome Assembly of Suaeda Glauca
Source: Front Genet. 2022 May 12;13:884081. doi: 10.3389/fgene.2022.884081 (PMC9135360; doi:10.3389/fgene.2022.884081)

## Slide 1
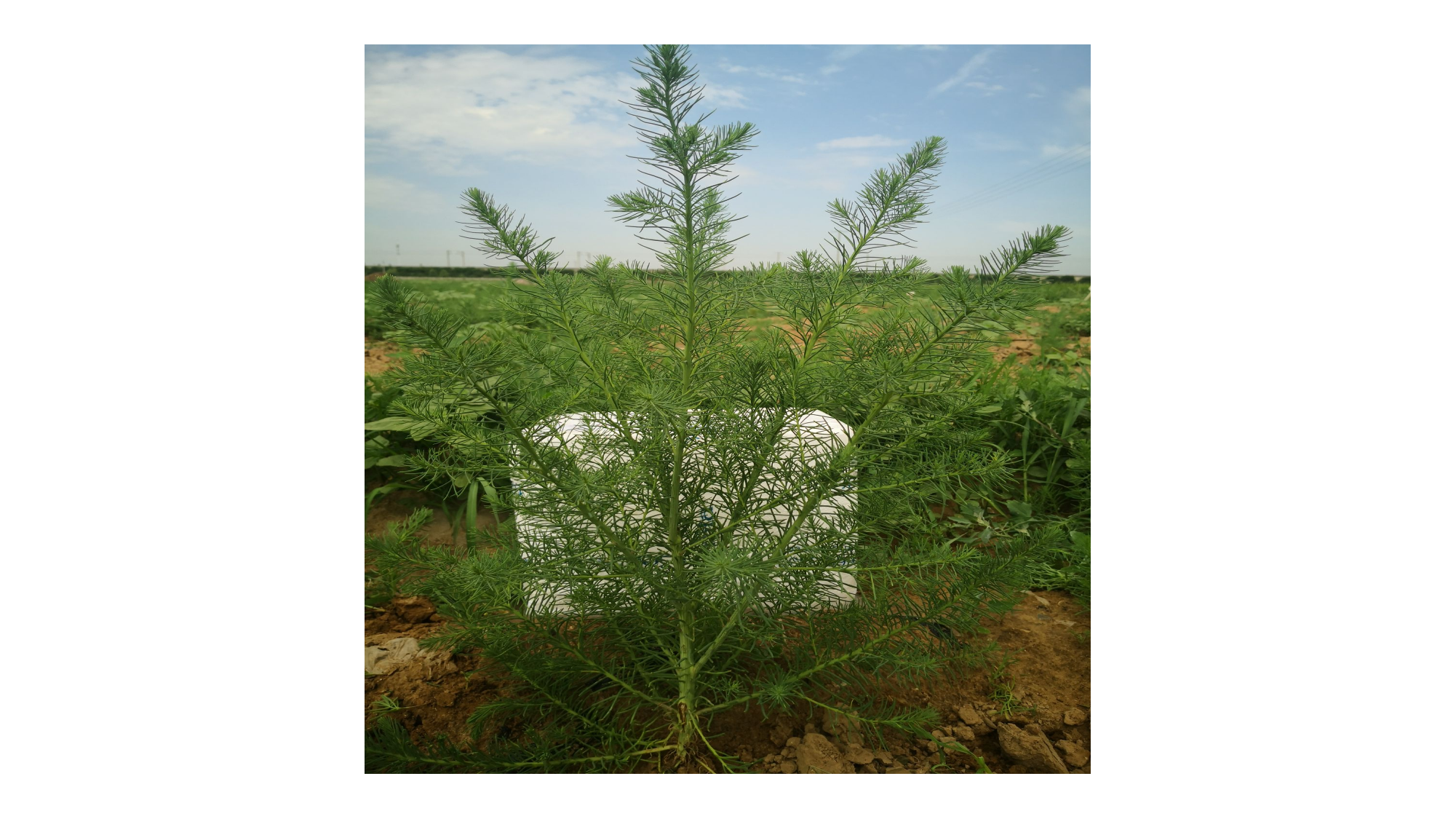

## Slide 2
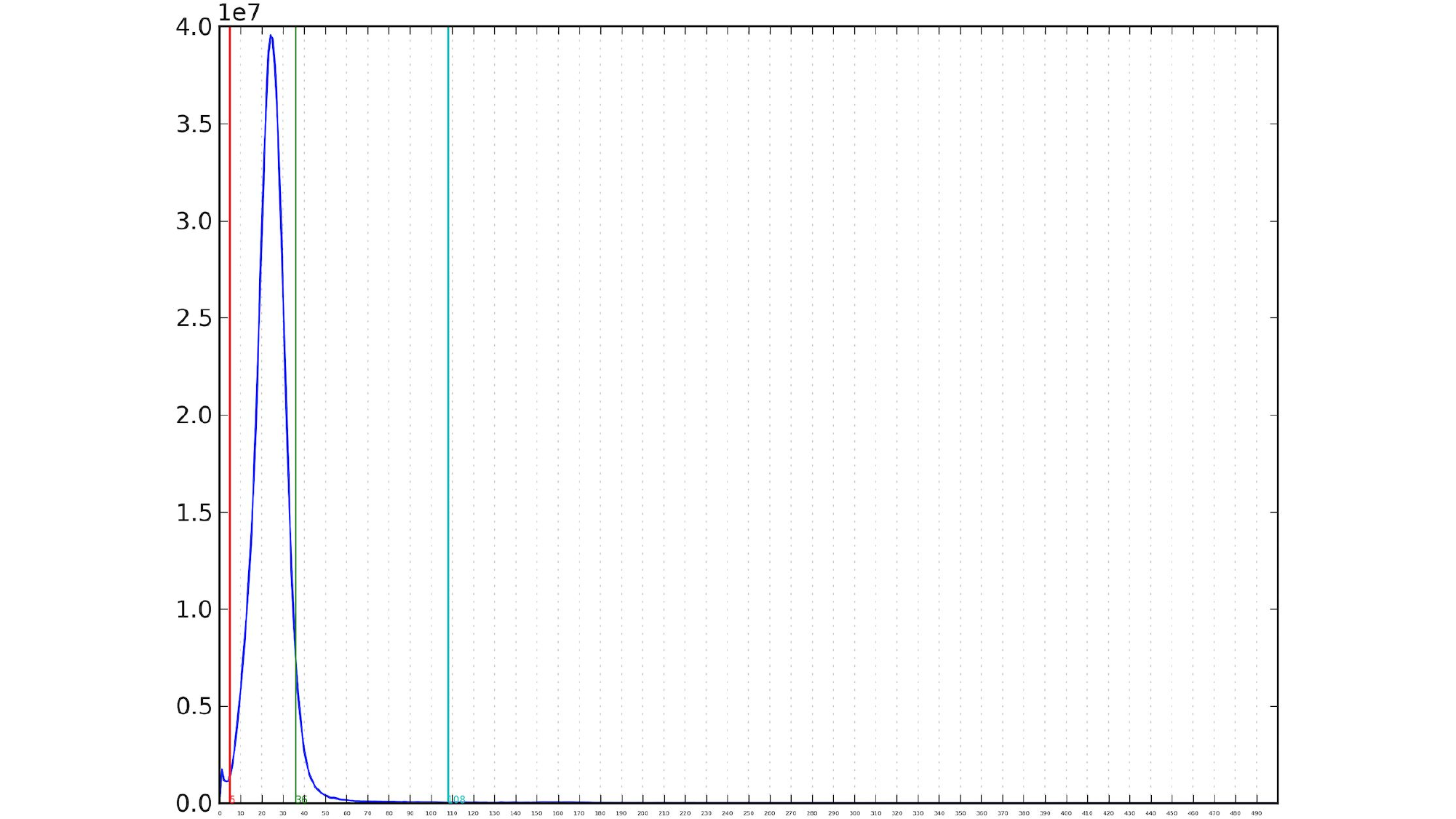

## Slide 3
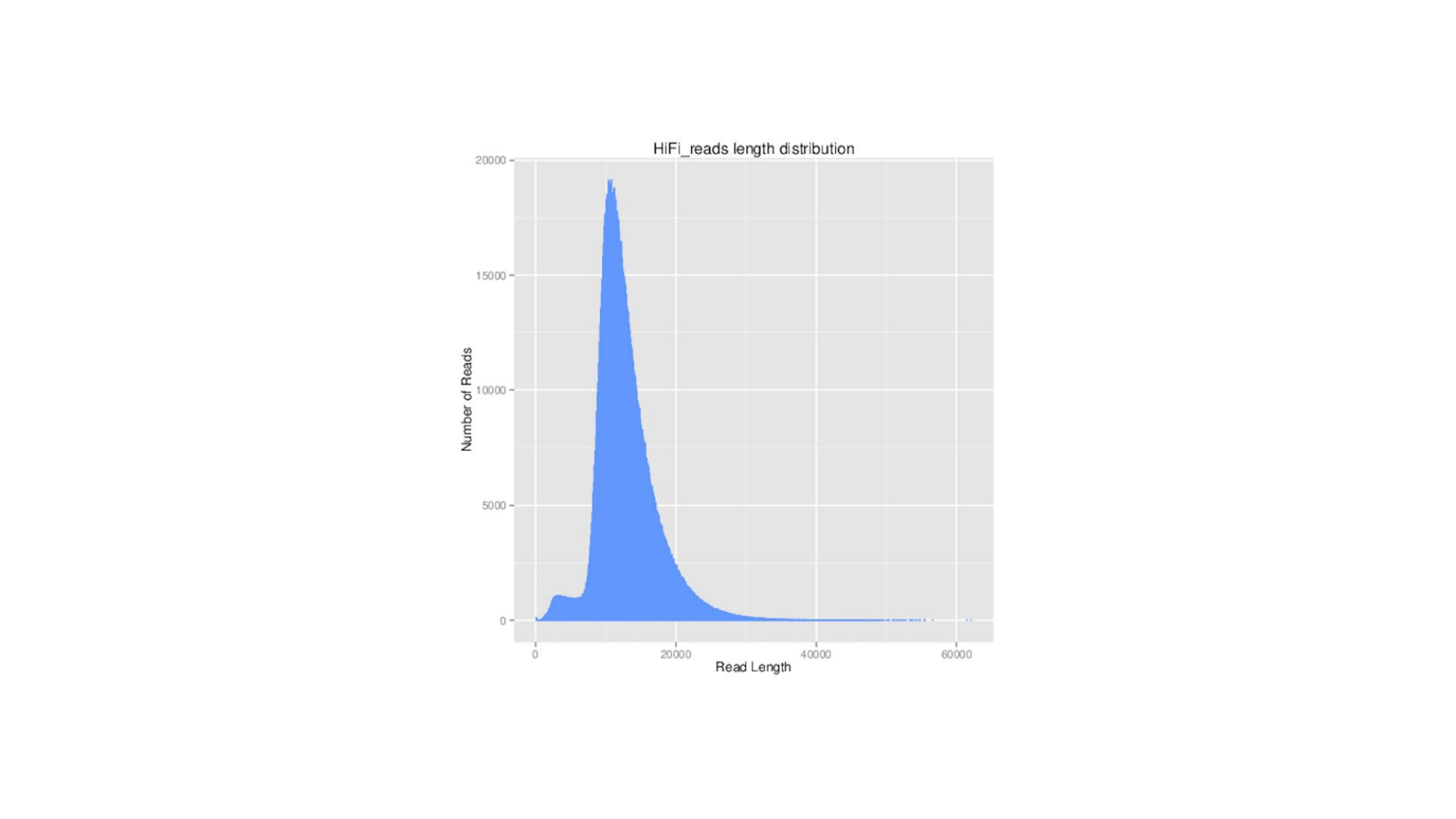

## Slide 4
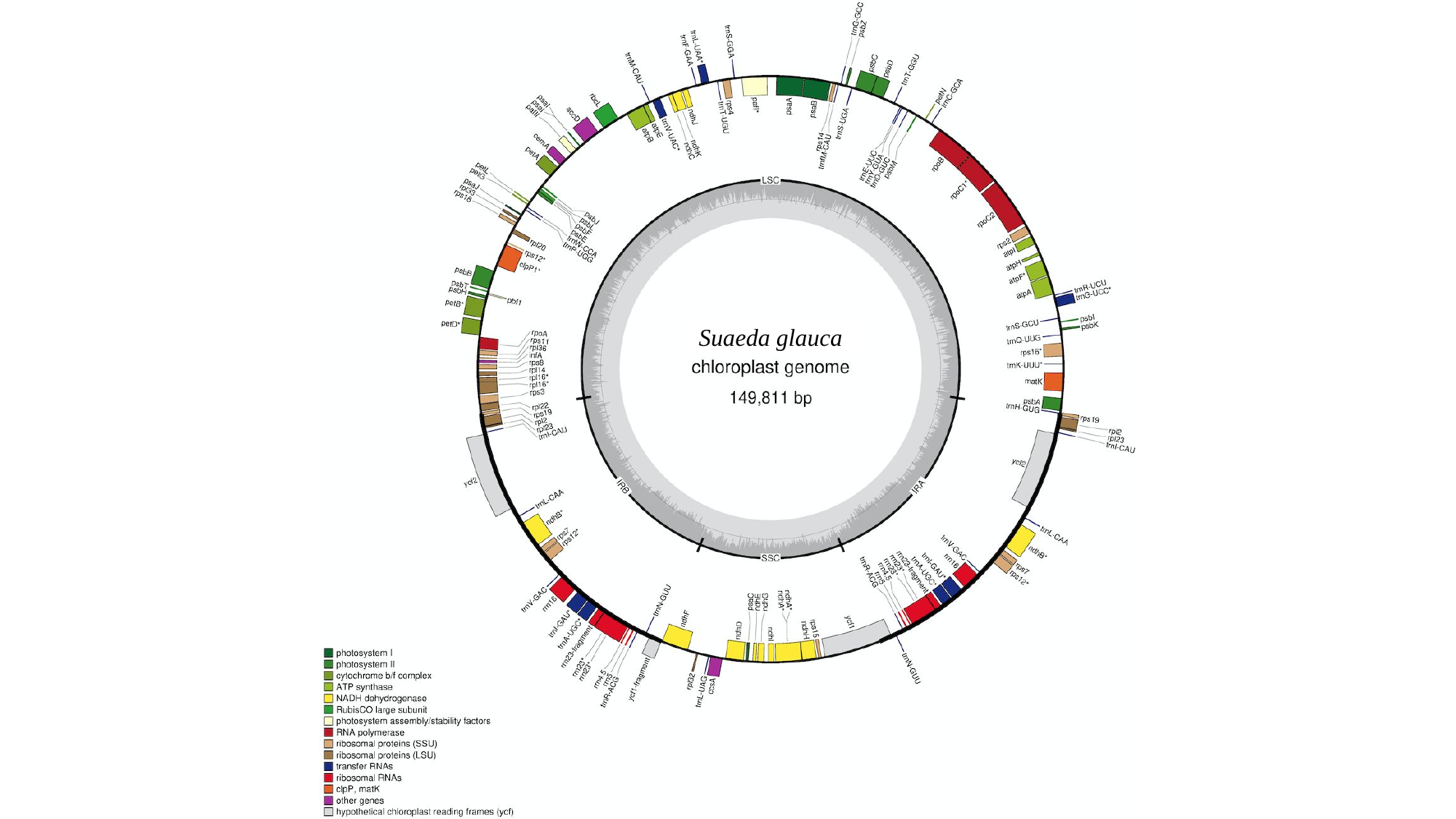

Suaeda glauca

## Slide 5
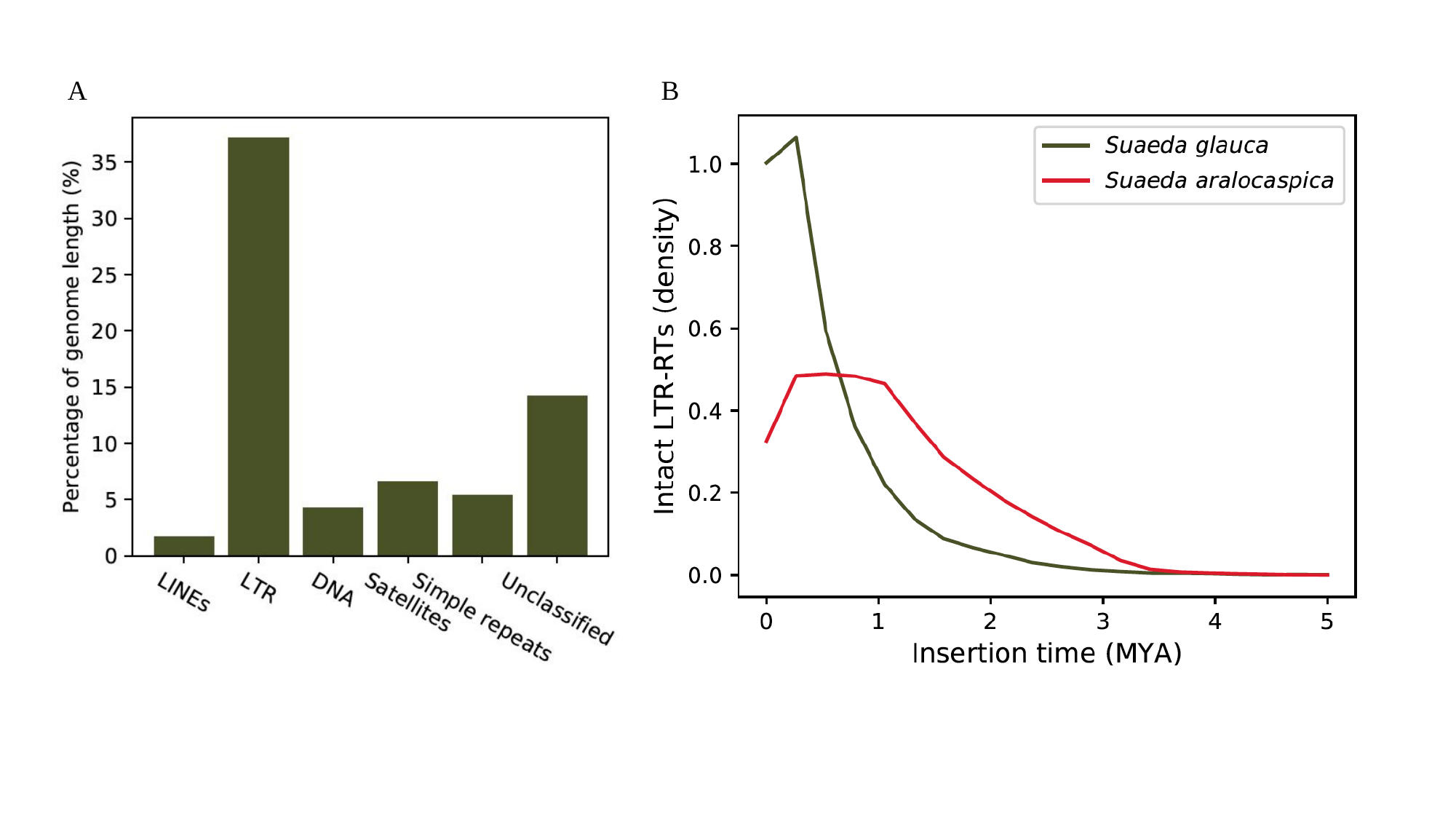

A
B

## Slide 6
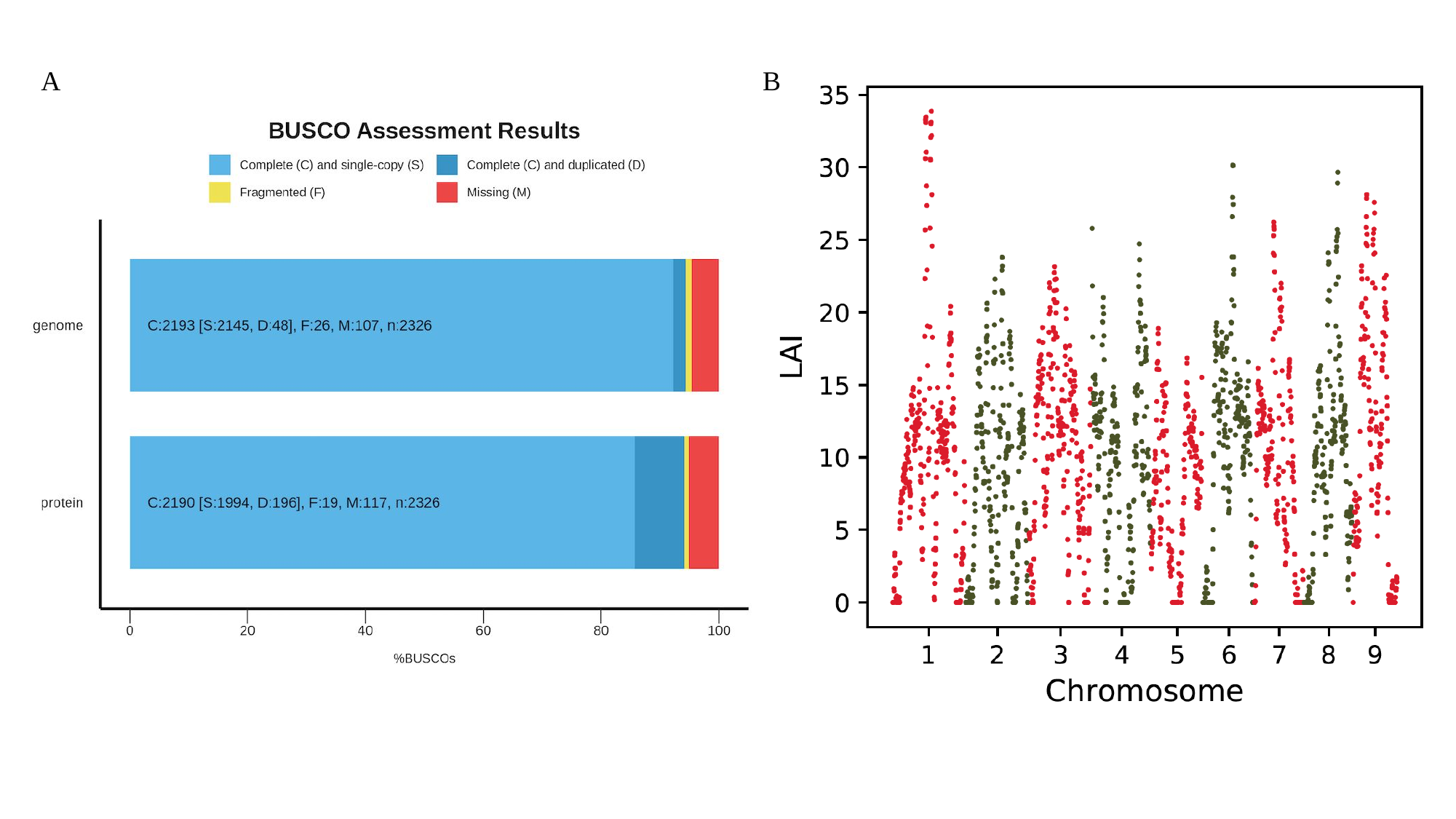

A
B

## Slide 7
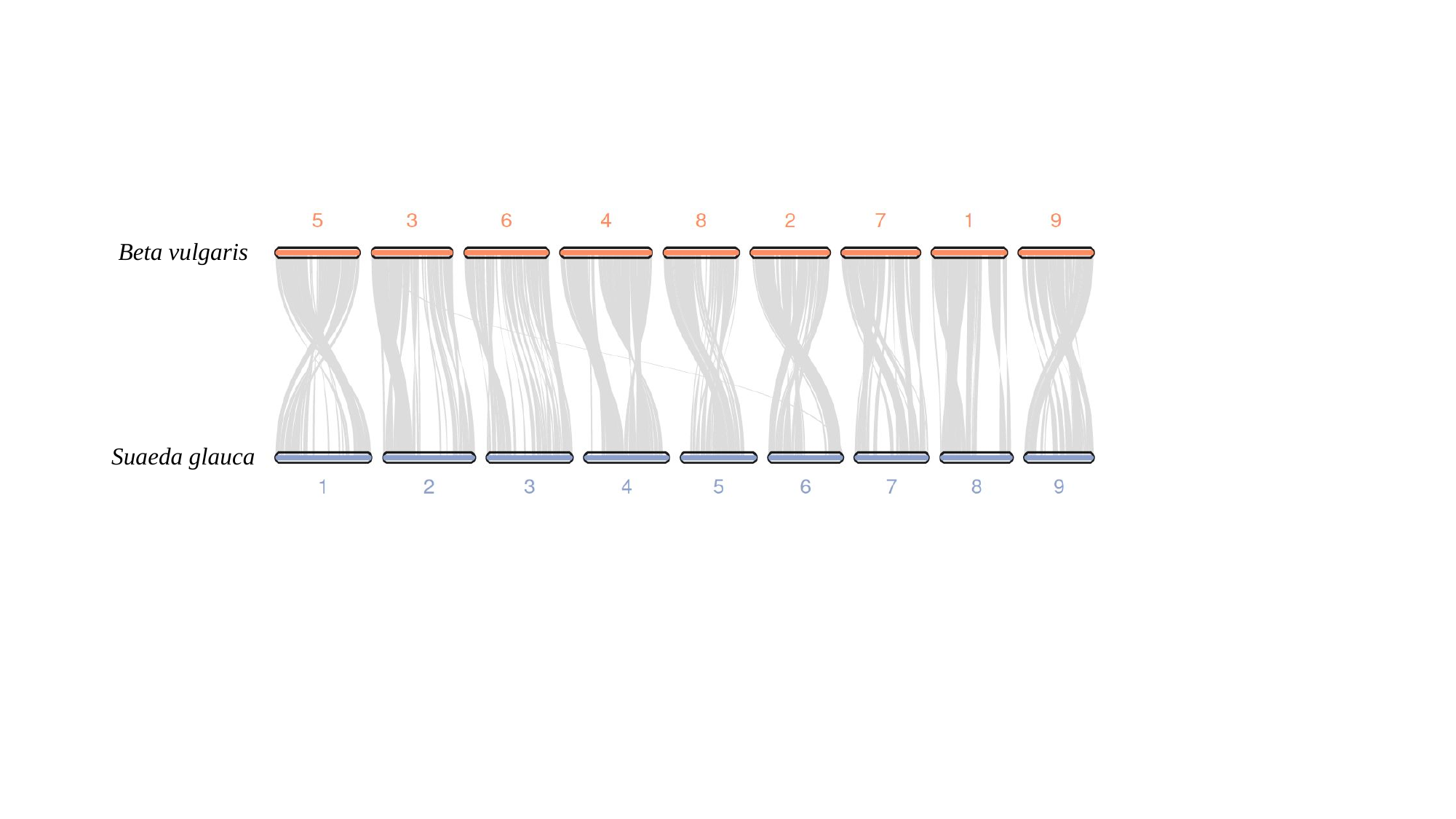

Beta vulgaris
Suaeda glauca

Supplement: Supplementary file 1 [file Presentation1.PPTX]
